# Suicide Prevention Intervention for Adults Presenting With Self-Harm in Pakistan: Cost-Effectiveness Analysis

**DOI:** 10.1192/bjo.2024.149

**Published:** 2024-08-01

**Authors:** Mohsin Hassan Alvi, Tehmina Ashraf, Nadeem Gire, Nasim Chaudhry, Nusrat Husain

**Affiliations:** 1 Pakistan Institute of Living and Learning, Karachi, Pakistan; 2 The University of Manchester, Manchester, United Kingdom; 3 University of Bolton, Bolton, United Kingdom

## Abstract

**Aims:**

Suicide is a serious global public health concern. Most suicide related deaths occur in low- and middle-income countries (LMICs) such as Pakistan. Self-harm is a major predictor of death by suicide and has cost implications both in terms of treatment and subsequent suicide. Therefore, culturally relevant interventions that have the potential to reduce self-harm in Pakistan may have major implications for the costs incurred by service provision and productivity losses due to illness or premature death. This trial investigated the clinical and cost effectiveness of the CMAP intervention verses enhanced treatment as usual (E-TAU) to reduce self-harm over 12 months in Pakistan.

**Methods:**

Participants were recruited from emergency departments, primary care settings, medical units of participating hospitals and self-referral from community settings in Karachi, Lahore, Rawalpindi, Quetta and Peshawar. Eligible consented participants were assessed at baseline, 3- (end of intervention), 6-, 9- and 12-month post-randomization. Participants in the intervention arm received 6 one-to-one sessions of culturally adapted manual assisted psychological intervention (CMAP) over 3 months. The Client Service Receipt Inventory was used to record health service utilization, both formal and informal. Health related quality of life was measured using the EQ-5D-3L. The Thailand tariff value set (developed by the EuroQol Organization) was used to calculate quality-adjusted life year (QALY) because Thailand was deemed similar to Pakistan. The Incremental Cost Effectiveness Ratio (ICER) was calculated based on between arm differences in estimated cost and Quality Adjusted Life Years (QALYs) gains in the sampled population. Costs were converted to US dollars using the currency exchange rate on February 2024 (US$1 = PKR276)

**Results:**

A total of 901 participants were randomized into either the CMAP arm (n = 440) or E-TAU arm (n = 461). Total QALY gained in the CMAP arm was 0.40 (95% CI: 0.36–0.45) and in the E-TAU arm was 0.33 (95% CI: 0.30–0.38) at 12-month post-randomization. The additional QALY gained due to CMAP intervention is 0.07. The difference in costs per participant between CMAP and TAU arms was US$59. The ICER for the CMAP versus E-TAU was US$843 per QALY gain.

**Conclusion:**

Results revealed that the CMAP intervention is likely to be cost-effective compared with the E-TAU, given the cost-effectiveness threshold. These findings suggest that implementing culturally relevant self-harm and suicide prevention measures such as CMAP can lead to significant societal cost savings by preventing self-harm and suicides.

